# South Korean Early Cancer Patients’ Perceptions of Difficulties in Fighting Their Disease: A Q Methodological Approach

**DOI:** 10.3390/ijerph191912510

**Published:** 2022-09-30

**Authors:** Min-Jeung Shim, Song-Yi Lee

**Affiliations:** 1 Counseling and Coaching, Graduate School, Dongguk University-Seoul, 30, Pildong-ro 1 gil, Jung-gu, Seoul 04620, Korea; 2 Dharma College, Dongguk University, Seoul 04620, Korea

**Keywords:** early breast cancer, breast cancer patients, difficulties, perception, Q methodology

## Abstract

This study applied the Q methodology to explore breast cancer patients’ perceived difficulties in their fight against the disease. We used literature analysis and in-depth interviews and selected 162 statements for the Q population. Then, we chose 40 universal and representative statements for the Q samples from the Q population. The P sample included 13 breast cancer patients in the early stage of the disease who participated in the Q sorting. We interviewed the study participants with high factor weights by type of P sample. The study’s results showed three types of breast cancer patients’ perceptions of difficulties in the initial fight against the disease. Type 1 showed ‘fear of the future’, Type 2 showed ‘helplessness against what cannot be controlled’, and Type 3 showed ‘frustration due to difficulties in role performance.’ Based on these results, we discuss the characteristics, meanings, and significance of individual types of breast cancer patients’ perceptions of the disease, including suggestions for follow-up studies.

## 1. Introduction

Globally, breast cancer is women’s leading cause of death [1]. The number of women diagnosed with breast cancer in South Korea has continually been on the rise, observing a 2.7% increase rate over the last two decades [2]. In the United States, one out of every eight women is diagnosed with breast cancer [3]. In addition, breast cancer is a major cause of mortality and morbidity in Middle Eastern countries [4]. As such, breast cancer is not only a problem for individuals but also a serious public health concern [5].

Early breast cancer patients who receive their diagnosis unexpectedly experience strong emotions, such as anxiety, anger, and resentment, due to the surprise of their sudden detection [6]. In addition, they may experience despair because breast cancer requires a treatment period of five to ten years, unlike other curable cancers within five years after surgery [7]. In particular, early breast cancer patients recognise the deformation of their breast as the biggest shock [8]; they endure pain in the reconstruction process and a sense of loss of their breast.

In addition, the difficulties experienced by breast cancer patients are considerable. They experience deterioration of their physical strength while undergoing treatments, such as chemotherapy, radiation therapy, and taking hormonal drugs. They also experience loss of hair and nails due to complications and side effects associated with cancer treatments [9,10]. Furthermore, they may have problems doing household chores or working at their workplaces due to physical pain [11]. Early breast cancer patients experience various stressors as their overall quality of life deteriorates [12,13].

Unfortunately, it is problematic that there is little psychological support available for patients who have survived cancer despite their worrisome fear of cancer recurrence [14]. Therefore, as women overcome breast cancer, long-term emotional factors become more influential during and after treatment [15]. Women with breast cancer also have lower sexual quality of life due to negative body image, physical inactivity, depression, sleep problems, chest symptoms, and chronic fatigue [16]. Therefore, their quality of life should include consideration of their psychological, physical, and living characteristics [11].

The longer the duration of breast cancer, the greater the stress [17]. This impact means that reducing stress in the long term through appropriate responses is essential in the initial fight against the disease. Although they experience such difficulties, individuals may perceive the characteristics and degree of problems they feel differently; an individual’s culture or society affects their subjective perception. The diagnosis and treatment of breast cancer are affected by social, economic, and cultural factors. For example, conservative views on femininity and women’s roles dominate Korean culture [18,19]. As such, the characteristics of difficulties experienced by female patients with breast cancer may differ in Korea compared to a Western nation. Therefore, early breast cancer patients need individual psychological and emotional support according to their cultures.

Individualised treatment is vital for breast cancer patients [20]. Thus, determining appropriate solutions requires identifying the individual’s subjective difficulties. Therefore, this study examined the subjective difficulties experienced by early breast cancer patients in South Korea to provide fundamental data that can help them overcome the disease.

## 2. Study Method

### 2.1. Study Procedure

The Q methodology is a scientific method for analysing individuals’ subjective perceptions through a statistical process. The methodology incorporates a qualitative and quantitative study approach, which are mutually complementary [21,22]. The study procedure involves selecting Q samples for representative and meaningful items based on the Q population; the Q samples are individuals’ subjective statements on the study topic. In addition, the procedure includes the selection of P samples, which are study participants, classification of Q samples, data processing, and analysis.

In the Q methodology, an objective analysis of subjectivity consists of factor analysis, analysis of correlations with factors, and profiling factors’ characteristics [23]. In this case, the factors produced through factor analysis indicate groups of individuals with similar responses [24]. In this study, referring to the types described previously [25], the researchers determined the number of factors as 1–10, executed the QUANL program, and then established the type with the highest explanatory power.

### 2.2. Selection of the Q Population and Q Samples

The Q population is the most crucial part of the Q methodology; it represents the general idea on the topic and comes from literature and interviews [26]. In this study, we selected 41 documents from academic journals and dissertations by entering ‘breast cancer’, ‘breast cancer survivors’, and ‘breast cancer patients’ as keywords. The selected examples included ‘influence of lifestyle, depression, and marital intimacy on quality of life in breast cancer survivors’ [27] and ‘living after diagnosis of middle-aged and elderly breast cancer survivors at early stages’ [28]. Next, we organised the contents of the selected studies to make statements. In addition, we used semi-structured open-ended questions to interview three breast cancer patients treated for 3, 6, or 11 months after breast cancer surgery. Examples of interview questions included “What difficulties do you experience while fighting breast cancer,” “What made you worry while fighting breast cancer,” and “What did you think while fighting breast cancer?” Based on the participants’ responses, our Q population comprised 162 statements.

Q samples refer to comprehensive and representative statements related to the topic in the Q population. The size of the Q samples was 40–60, which is the best according to experience [29]. We selected Q samples from the Q population through three processes in this study. In the first process, classification, one breast cancer patient who underwent breast cancer surgery three months earlier and one Q methodology researcher selected 78 Q samples by removing items with overlapping or identical meanings. In the second process, two Q methodology researchers determined the Q samples. Finally, in the third process, one breast cancer patient and two Q methodology researchers selected Q samples to specify the final 40 questions. Table 1 shows the selected Q samples.

### 2.3. P Sample Selection

The P sample is the Q participants. This group will proceed to Q sorting and determine the characteristics of perceptions by type [30]. Since the Q methodology does not aim at generalisation, samples in the range of 10–100 persons are suitable for the P sample following the small sample principle [29,31]. In this study, we used the purposive sampling method to select 13 patients with early-stage breast cancer treated with anti-cancer chemotherapy, radiation therapy, etc., less than a year after total or partial mastectomy.

### 2.4. Q Sorting

We conducted Q sorting as follows. First, 13 study participants read the statements of 40 Q samples and placed them on the Q sample distribution chart in the form of a normal distribution depending on the relative meanings and importance of the statements. We explained the study’s purpose, meaning, and contributions to the participants, who then went through a confirmation procedure before agreeing to participate. We conducted Q sorting for 18 days from 12–30 January 2022.

**Figure 1:**
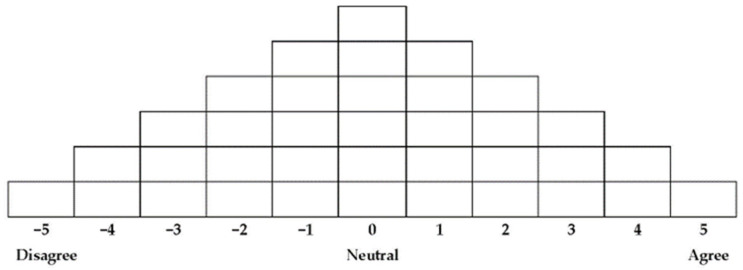
Q sample classification table.

In Q-sorting, the study participants read the statement cards and sorted them into three groups (agree, neutral, and disagree) per their subjective perceptions. Then, they placed the statement cards with which they agreed most strongly (+5) on the rightmost side and those they disagreed with most strongly (−5) on the leftmost side. Next, we asked the participants to describe their reasoning for selecting the two statements at either end (i.e., strongly agree and strongly disagree). We used these descriptions as data to analyse the characteristics by type. The total time required for Q sorting was 30–45 min; Figure 1 shows the Q sorting distribution.

### 2.5. Q Sorted Data Processing and Analysis

We used the statement scores by item in the 1–11 points range for data analysis. Then, we converted these scores based on the raw scores of −5 points (*strongly disagree*) to +5 points (*strongly agree*) in the Q sort sheets, which are the data Q-sorted by the 13 participants (i.e., the P sample). After that, we conducted a principal component factor analysis with the data using the QUANL program to produce factors and utilise Z-scores [29]. Table 2 is the score conversion table.

Following this, we interviewed the study participants with high factor weights by type from 10 February to 22 February 2022. We used the results to interpret each type along with the study findings. This approach allowed us to learn more about each type’s characteristics based on this study’s results. The study participants who participated in this interview were P12 for Type 1, P13 for Type 2, and P2 for Type 3.

## 3. Study Results

### 3.1. Analysis of Results

We analysed the early breast cancer patients’ types of perceptions of difficulties and classified their perceptions into three types (Table 3). The eigenvalues of individual types were 3.4018 for Type 1, 1.3569 for Type 2, and 1.1593 for Type 3, and the cumulative variance (explanatory power) was 0.4552.

The correlations between individual types show the degree of similarity between types. In this study, the correlation coefficient between Types 1 and 2 was 0.291, Types 1 and 3 was 0.249, and Types 2 and 3 was 0.128 (Table 4).

### 3.2. Characteristics by Type

Table 5 shows the factor weights for the types of perceptions of difficulties among early breast cancer treatment patients. The higher the factor weight of a type, the more representative that relevant type. In this study, the representative persons (P samples) for individual types were P12 (factor weight of 1.3366 for Type 1), P13 (factor weight of 1.6403 for Type 2), and P2 (factor weight of 1.0198 for Type 3).

#### 3.2.1. Type 1: Fear of the Future

As presented in Table 6, among the Type 1 respondents’ statements regarding difficulties in the initial fight against breast cancer, major statements that garnered strong agreement included Q28 ‘I am afraid that my breast cancer might spread or that I might have complications’ (Z = 2.28) and Q29 ‘I am afraid that my breast cancer might come back’ (Z = 1.89).

P6, who best showed the characteristics of Type 1, said, “Although currently, the cancer mass has been removed by total resection because my breast cancer is in the early stage, I am afraid that the cancer may metastasise so that I cannot work due to treatment from anti-cancer therapy to surgery eventually leading to the collapse of my daily life and my family members”.

In addition, P5 showed fear about the future with the expected anti-cancer treatment, stating, “I’ve read a lot of articles indicating that breast cancer has nothing to do with stages. I am afraid because it is said that when it has metastasised, it begins with stage 4 immediately, and surgery for it cannot be performed immediately, and drugs for it cannot be easily found. I am nondescriptly afraid and scared because I have not yet seen a patient who has been cured in stage 4. I don’t know how to treat it, and it seems like it’s still going on”.

Furthermore, P12 said, “I feel like I am living with death on my back. No one knows what will happen to me tomorrow. I’ve been planning step by step and working hard so far, but now I think I cannot see beyond my nose. I am really frustrated and afraid of how I should live in the future”.

#### 3.2.2. Type 2: Helplessness against What Cannot Be Controlled

Table 7 presents the Type 2 respondents’ statements about difficulties in the initial fight against breast cancer. Major statements with which respondents strongly agreed were Q1 ‘I feel helpless because cancer is a disease that is beyond my control’ (Z = 2.32) and Q26 ‘I am worried about chemotherapy treatment because of its possible side effects that can lead to other illnesses’ (Z = 1.54).

P11, who best showed the characteristics of Type 2, said, “I think I feel inexplicit fear and feel that all the treatment processes are difficult because I have no existing information since I have never imagined or experienced a cancer diagnosis”.

In addition, P3 was embarrassed with her current unexpected situation, stating, “I used to think that I was taking good care of my health at normal times, but after I was diagnosed with cancer, I have been worried about how I should manage it hereafter”.

In addition, P13 said, “I think I’ve done everything that is good for my health so far. I did my best by focusing on my health in terms of food and exercise, but I got cancer. I don’t know how to live a better life than before”.

#### 3.2.3. Type 3: Frustration Due to Difficulties in Role Performance

As presented in Table 8 among the Type 3 respondents’ statements about difficulties in their initial fight against breast cancer, major statements with strong agreement were Q36 ‘I am worried that I might not be able to work anymore’ (Z = 1.55) and Q19 ‘I am worried about not being able to fulfil my role and responsibilities at home during treatment’ (Z = 1.54).

P8, who best showed the characteristics of Type 3, said, “My job has been good as I felt a sense of accomplishment because it required thinking, planning, putting my aims into practice, and producing results, but I cannot obtain as many results or as great a sense of accomplishment as before, although it has been said that I should not feel hard and this seems to undermine the driving force in my life”.

In addition, P7 indicated her loss and difficulties in her current roles in her family and workplace, stating, “Last Lunar New Year’s holidays when less than two weeks passed after my surgery, I went to the countryside and prepared all things for the holidays. Since I found it hard to move throughout the period when I was undergoing chemotherapy, I crawled to do all my household chores and prepare meals”.

Furthermore, in her interview, P2 said, “I have a huge appetite for work; the company recognised my abilities and thus raised my salary, and the CEO gave me a lot of support because he knew about my efforts. When I stopped working because I was sick, the CEO told me to come back when I’m well, but now I don’t think I can go back”.

As presented in Table 9, there were two common statements, Q1 and Q19.

## 4. Discussion

This study found three types of perceptions about the difficulties experienced by early breast cancer patients: Type 1, ‘fear of the future’, Type 2, ‘helplessness against what cannot be controlled’, and Type 3, ‘frustration due to difficulties in role performance’.

Type 1 patients feared the uncertain future in which breast cancer metastasis, recurrence, etc., might occur. According to studies [32,33], breast cancer patients’ fears of the future are a vital issue, supporting our study’s characteristics of Type 1. However, these patients do not fear changes in their appearance. Intolerance of uncertainty and worry is a psychological factor that causes fear of cancer recurrence (FCR) in early-stage breast cancer survivors in clinics [34]. This characteristic corresponds to Type 1; therefore, addressing this aspect of FCR could support Type 1 patients.

Cognitive behavioural therapy related to positive psychology benefits breast cancer patients by providing appropriate coping measures for their fight against the disease [35]. Moreover, breast cancer patients who received positive psychotherapy or cognitive behavioural stress management assistance experienced a decrease in psychological distress, which had a positive effect as breast cancer recurrence decreased [36]. This finding indicates that Type 1 patients need psychological support to help them manage their difficulties.

Type 2 patients were embarrassed to have breast cancer despite managing their health carefully. However, they did not experience significant difficulties concerning loss, humiliation in relationships, or others’ prejudices. They appeared to be aware of the challenges since they could not control their lives. A study by Hernandez et al. [37] found that cancer patients feel lethargic, similar to the difficulties experienced by Type 2 in our study. Thus, they need greater capacity for self-efficacy, which is a crucial element for breast cancer patients in fighting the disease [38]. Self-efficacy is the self-judgement that one can solve problems by taking appropriate actions in one’s circumstances with belief in one’s abilities [39]. Enhancing self-efficacy, a positive self-concept will enable early breast cancer patients to control their emotions and thoughts and take the best concrete and practical actions to recover their health in their fight against cancer.

Type 3 patients feel discouraged that their role performance at home or in the workplace has changed since they developed breast cancer. However, the results show that they did not think that their family members who wanted them to perform their role identically to what they did before they developed breast cancer were selfish. Spousal support did not affect breast cancer patients’ quality of life [40]. Rather, they may need support and help from other breast cancer patients with the same experiences more than spousal support. Patients with similar experiences can help breast cancer patients overcome the difficulties associated with battling the disease.

Another study found that many women with breast cancer want to help other patients and feel helpful [41]. In addition, according to Chin et al. [42], mothers with breast cancer need support to improve intimacy in their mother–child relationships while preparing for an uncertain future. This finding means that, like Type 3, patients need support to continue their emotional role as mothers at home. Moreover, being supported by colleagues at work during cancer treatment stands out as a vital and lasting protective factor for continued work capacity [43]. Therefore, communication with colleagues at work is an important aspect of their fight against the disease.

Statements commonly shown by all types included Q1 ‘I feel helpless because cancer is a disease beyond my control’ and Q19 ‘I am worried about not being able to fulfil my role and responsibilities at home during treatment.’ These results show that it is essential for patients to live independently and proactively. In addition, to improve breast cancer patients’ quality of life, education and family support that enable patients to develop knowledge about breast cancer are crucial [44]. They commonly need to objectively accept situations they cannot control and understand that, instead of being caregivers, they are subjects that can receive care while they fight the disease.

The results of this study led to some suggestions as follows. First, breast cancer patients must communicate their difficulties in fighting the disease. In particular, early breast cancer patients should continuously communicate with their family members and the people in their lives to have an objective view on their situation. For instance, a study by Zhang et al. [45] found that breast cancer patients do not communicate with their children about their breast cancer, even in family relationships. Furthermore, since they are uncertain about their future, they are also uncertain when and how to tell their children about their cancer. However, our study found that it is crucial for breast cancer patients to understand the necessity of communication and an appropriate method.

Second, not all early-stage breast cancer patients suffer from the same difficulties. For example, Table 6 shows that Type 1 patients fear breast cancer recurrence but do not perceive significant challenges due to changes in their bodies or physical fatigue appearing during treatment. Although they experience difficulties fighting the disease, they do not experience all the difficulties breast cancer patients may experience. Therefore, it is necessary to identify each breast cancer patient’s challenges and provide support measures accordingly.

Third, although this study examined breast cancer patients’ perceptions of the difficulties they experience, the study’s results show that one should note the strengths they demonstrate in overcoming their perceived obstacles. It is also necessary to pay attention to the positive characteristics underpinning individual patients’ difficulties to overcome them.

Early breast cancer patients experience various physical, psychological, and social disabilities. Therefore, adaptability to change in multiple areas of life is essential, and they urgently need different countermeasures [46,47,48]. Finally, this study provides the basis for policy support, such as programs, education, and training to improve the adaptability of early breast cancer patients.

## 5. Conclusions

This study found three types of perceptions of difficulties experienced by early-stage breast cancer patients. However, the characteristics of their challenges differ. Therefore, each type requires appropriate support methods to fight the disease.

## 6. Limitations

This study examined the subjective perceptions of a few research participants using Q-methodology. This methodology is appropriate for identifying the types and characteristics recognised by early breast cancer patients in this study but does not allow for the generalisability of the study’s results. Moreover, we could not examine the difficulties of breast cancer patients in terms of their demographic backgrounds. Quantitative studies on how the perceptions of difficulties change depending on the age of breast cancer patients are warranted.

In addition, this study focused only on female breast cancer patients. Therefore, future research should also investigate male breast cancer patients’ perceptions [35], even though there are fewer male than female patients. In addition, since the study participants were all married, we could not grasp the characteristics of the difficulties perceived by unmarried subjects. Future studies should elucidate such aspects. Furthermore, it is necessary to provide policy guidelines for promoting appropriate health for each type and research what they perceive as these guidelines.

## Figures and Tables

**Table 1:** Q samples.

No.	Statement
1	I feel helpless because cancer is a disease that is beyond my control.
2	I feel flustered when I cannot control my emotions, leading me to have sudden feelings of depression and anxiety.
3	I always feel exhausted from lack of sleep due to continual worry about having breast cancer.
4	I feel lonely and isolated.
5	I am angry because I feel it is unfair that I got breast cancer.
6	Not knowing when I will die fills me with fear.
7	I feel pathetic when I see myself trying so hard not to feel anxious.
8	I feel helpless because I cannot plan for my future.
9	I feel like I am suffering from severe pain due to chemotherapy.
10	I feel doubtful about whether I will ever be cancer-free.
11	I feel a sense of loss about losing my breasts after undergoing mastectomy surgery.
12	I am afraid my partner will treat me differently regarding our sex life.
13	It is uncomfortable for me to go to public places like saunas.
14	I find it difficult to accept how I look due to hair loss.
15	I feel that I have lost my feminine identity.
16	I fear my nails falling off and my skin discolouring during treatment.
17	My family cannot understand my suffering.
18	I am worried that I will burden my family.
19	I am worried about not being able to fulfil my role and responsibilities at home during treatment.
20	I feel easily hurt when people around me try to console me with superficial greetings.
21	I am afraid that my breast cancer might be genetic.
22	I feel drained when people who had the same experience overwhelm me with excessive emotional expression and information.
23	I feel that my family and colleagues do not care when they still expect me to fulfil my roles and responsibilities.
24	I have resentment because I feel I got breast cancer due to continual stress from my family and work.
25	I do not want people to gossip about me being a breast cancer patient.
26	I am worried about chemotherapy treatment because of its possible side effects that can lead to other illnesses.
27	I feel that the immense amount of examinations I undergo at the hospital is burdensome and a hassle.
28	I am afraid that my breast cancer might spread or that I might have complications.
29	I am afraid that my breast cancer might return.
30	I feel that it is difficult to find a reliable surgeon.
31	It makes me sad when I think about the possibility of a day when I will have to accept that I will die because my breast cancer returned and I ran out of treatment options.
32	I feel degraded when medical professionals treat me without empathy, as if our relationship is strictly business.
33	I am frustrated because it is difficult to understand what the medical professionals say regarding my treatment process and the resultant symptoms.
34	It is difficult to accept that I no longer have a normal life.
35	I am worried that my coworkers will judge me and lower their expectations concerning my work ability because of my cancer.
36	I am worried that I might no longer be able to work.
37	I feel like everything I have built throughout my life has become meaningless.
38	I feel burdened about having to care more about my health.
39	I feel that treatment is too expensive.
40	I think that I will eventually have to end my career.

**Table 2:** Q-Sample classification and score construction.

Raw Score	−5	−4	−3	−2	−1	0	+1	+2	+3	+4	+5
Converted score	1	2	3	4	5	6	7	8	9	10	11

**Table 3:** Eigenvalues and explanatory variances in the classification of the three types.

Content/Type	I	II	III
Chosen eigenvalue	3.4018	1.3569	1.1593
Total variance	0.2617	0.1044	0.0892
Cumulative variance	0.2617	0.3661	0.4552

**Table 4:** Correlations.

	Type 1	Type 2	Type 3
Type 1	1.0000		
Type 2	0.291	1.000	
Type 3	0.249	0.128	1.000

**Table 5:** P sample and (P) weights.

Type	P	Gender	Age	Treatment Period (Months)	Marital Status	Occupational Status	Factor Weight
Type 1 (*n* = 7)	1	Female	50	7	Married	Housewife	0.4254
	4	Female	51	1	Married	Housewife	0.7596
	5	Female	43	2	Married	Housewife	0.7764
	6	Female	39	3	Married	Unemployed	0.8815
	9	Female	45	7	Married	Employed	0.5381
	10	Female	53	7	Married	Housewife	0.6871
	12	Female	54	3	Married	Employed	**1.3366**
Type 2 (*n* = 3)	3	Female	58	1	Married	Employed	1.0520
	11	Female	65	8	Married	Housewife	0.7990
	13	Female	53	11	Married	Housewife	**1.6403**
Type 3 (*n*= 3)	2	Female	41	8	Married	Unemployed	**1.0198**
	7	Female	52	11	Married	Housewife	0.1803
	8	Female	43	4	Divorced	Leave of absence	0.8183

Factor weight (bold): highest factor weight for each type.

**Table 6:** Representative statements and standard scores (at least ±1.00) of Type 1.

No.	Statement	Standard Score
28	I am afraid that my breast cancer might spread or that I might experience complications.	2.28
29	I am afraid that my breast cancer might return.	1.89
1	I feel helpless because cancer is a disease that is beyond my control.	1.84
36	I am worried that I might not be able to work anymore.	1.14
21	I am afraid that my breast cancer might be genetic.	1.10
37	I feel like everything I have built throughout my life has become meaningless.	1.02
11	I feel a sense of loss about losing my breasts after going through mastectomy surgery.	−1.94
16	I fear my nails falling off and my skin discolouring during treatment.	−1.58
3	I always feel exhausted from lack of sleep due to continual worries about having breast cancer.	−1.39
2	I feel flustered when I cannot control my emotions, leading me to have sudden feelings of depression and anxiety.	−1.26
14	I find it difficult to accept how I look due to hair loss.	−1.22

**Table 7:** Representative statements and standard scores (at least ±1.00) of Type 2.

No.	Statement	Standard Score
1	I feel helpless because cancer is a disease that is beyond my control	2.32
28	I am afraid that my breast cancer might spread or that I might have complications.	1.76
26	I am worried about chemotherapy treatment because of its possible side effects that can lead to other illnesses.	1.54
25	I do not want people to gossip about me being a breast cancer patient.	1.39
38	I feel burdened about having to care more about my health.	1.37
29	I am afraid that my breast cancer might return.	1.24
27	I feel that the immense amount of examinations at the hospital is burdensome and a hassle.	1.18
21	I am afraid that my breast cancer might be genetic.	1.05
37	I feel like everything I have built throughout my life has become meaningless.	−1.94
31	It makes me sad when I think about the possibility of a day when I have to accept that I will die because my breast cancer returned and I ran out of treatment options.	−1.78
35	I am worried that my coworkers will judge me and have lower expectations from me regarding my work ability because of my cancer.	−1.48
36	I am worried that I might not be able to work anymore.	−1.29
32	I feel degraded when medical professionals treat me without empathy, as if our relationship is strictly business.	−1.10

**Table 8:** Representative statements and standard scores (at least ±1.00) of Type 3.

No.	Statement	Standard Score
1	I feel helpless because cancer is a disease that is beyond my control.	2.10
36	I am worried that I might not be able to work anymore.	1.55
19	I am worried about not being able to fulfil my role and responsibilities at home during treatment.	1.54
40	I think that I will eventually have to end my career.	1.35
13	I find it uncomfortable to go to public places such as saunas.	1.24
18	I am worried that I will burden my family.	1.06
24	I have some resentment because I feel I got breast cancer due to the continual stress from my family and work life.	−2.02
6	Not knowing when I will die fills me with fear.	−2.01
21	I am afraid that my breast cancer might be genetic.	−1.99
3	I always feel exhausted from lack of sleep due to continual worries about having breast cancer.	−1.56
4	I feel lonely and isolated.	−1.54
23	I feel that my family and colleagues do not care when they still expect me to fulfil my roles and responsibilities.	−1.37

**Table 9:** Common statements.

No.	Statement	Standard Score
1	I feel helpless because cancer is a disease that is beyond my control.	2.09
19	I am worried about not being able to fulfil my role and responsibilities at home during treatment.	1.16

## Data Availability

Data from the study are available upon request.
